# Dynamic stratified porosity computation from canopy interaction simulation between airflow and leaves

**DOI:** 10.3389/fpls.2023.1238360

**Published:** 2023-10-18

**Authors:** Huiyuan Cui, Chengde Wang, Fadian Lu, Xuemei Liu, Jin Yuan

**Affiliations:** ^1^ College of Mechanical & Electronic Engineering, Shandong Agricultural University, Tai’an, China; ^2^ Forestry College, Shandong Agricultural University, Tai’an, China; ^3^ Shandong Provincial Key Laboratory of Horticultural Machinery and Equipment, Tai’an, China

**Keywords:** stratified porosity, CFD, image processing, fluid-structure interaction, leaf deformation

## Abstract

The main goal of wind-driven spraying is to use assisted airflow to disrupt the structure of branches and leaves and broaden the air delivery channel, so as to achieve uniform droplet deposition in the middle and lower parts of the canopy. Due to the complex branch and leaf structure inside the canopy, there is currently no effective method to express the dynamic changes of canopy porosity and the law of airflow attenuation under assisted airflow. In this study, based on the two-way fluid-structure interaction numerical simulation method, the relating between the assisted airflow and the structural parameters of the cotton canopy is analyzed, and a new method for predicting and simulating the dynamic porosity of the canopy is proposed. Firstly, a two-way fluid-structure interaction model based on *Lattice Boltzmann* (LB) solver and *Finite Element* (FE) solver is developed to simulate the deformation motion of cotton leaves and the spatial distribution of airflow field, and the correctness of the numerical simulation is verified based on indoor measurement data. Secondly, the post-processing method of Computational Fluid Dynamics (CFD) is used to obtain images of leaves at different canopy positions under assisted airflow, and the porosity changes are calculated and analyzed by image processing. The research results show that under different initial wind speeds (5 m·s^-1^, 10 m·s^-1^, 15 m·s^-1^), the maximum normalized mean absolute error (NMAE) between the simulated values and the measured values is 13.99%, 20.72% and 16.08%, respectively. The coefficient of determination (R^2^) for linear fitting between simulated values and measured values is 0.9221. These validation results indicate the effectiveness of the numerical simulation method. The validated CFD model is applied to predict leaf deformation and porosity changes within the canopy under various wind loads and times. The application results have well revealed the interaction between crop leaves and airflow, and will be beneficial to make a better understanding of the effect of assisted airflow on droplet deposition.

## 1 Introduction

In the process of plant protection application, a large amount of pesticide misapplication will reduce the effectiveness of pesticide application and increase environmental pollution (Gil and Sinfort, 2005; Damalas and Eleftherohorinos, 2011; Tudi et al., 2021). The main goal of precision pesticide application is to achieve uniform coverage and deposition of pesticides in the target crop canopy (Khot et al., 2012; Li et al., 2021; Grella et al., 2022). The canopy characteristics of target plants directly affect the application mode and the droplet deposition effect (Duga et al., 2015; Xun et al., 2022). A complete understanding of the canopy characteristics of the target plant is important to evaluate the airflow velocity and turbulence levels within the canopy.

Canopy parameters are not only important indicators of growth and yield, but also important factors affecting pesticide interception and deposition (Olesen et al., 2003; Liu et al., 2020). In the late stage of crop growth, the stems and leaves of the plant population cover each other. Air-assisted sprayers can effectively deliver pesticides within dense canopies (Reichard et al., 1979; Derksen et al., 2008). The disturbance of the assisted airflow on the canopy branches and leaves can change the porosity of the canopy, thus widening the transmission channel of the pest control agent, which helps to achieve the droplet deposition at the lower canopy (Müller et al., 2018; Qiu et al., 2022). Scholars have conducted a large number of spray deposition field tests and wind tunnel tests on different target crops with different air-assisted sprayers.

Cross et al. (2001a, 2001b) conducted a series of field experiments on apple trees of different sizes to study the complex interaction between air-volume flow rate, spray-liquid flow rate, spray quality (droplet size distribution) and crop characteristics. Chen et al. (2013a; 2013b) measured the spray retention and non-target deposition at three crown growth stages (i.e. leaf stage, half leaf stage and full leaf stage), and found that increasing canopy density significantly reduced the amount of drift from the target. Duga et al. (2015) analyzed the spray deposition profiles in different pome fruit trees and concluded that tree characteristics such as total leaf cover, leaf wall porosity and tree volume strongly influenced total on-target deposition. These empirical spray studies indicate that spray deposition is caused by the complex interaction between the canopy and the air in canopy.

Crop spraying is a complex process involving the interaction of many parameters, such as pesticide dose and spray volume, spray-liquid distribution, droplet spectrum, air volume, sprayer speed, meteorological conditions and crop characteristics. In the past few decades, modeling approaches, especially computational fluid dynamics (CFD) models, have been effectively used to understand and characterize the crop spraying process (Badules et al., 2018; Zhang et al., 2018). Scholars adopted the averaging procedure to model airflow within a plant canopy without considering the flow details of individual elements (Wilson and Shaw, 1977; Raupach and Shaw, 1982). The canopy is considered as a porous medium to study the transport of airflow and droplets in canopy. The properties of porous medium are determined by the structural parameters of the canopy (porosity, leaf density) (Da Silva et al., 2006; Cui et al., 2022; Zhang et al., 2022). Endalew et al. (2009), 2010 simulated the effect of large branches and airflow by adding a resistance term in the canopy. The leaves in the canopy have a large effect, especially on the crop canopy. Dorr et al. (2008) developed a turbulence probability model, which can combine the motion model of fog droplets with the three-dimensional structure of plants, and simulate the drift of pesticide droplets around different plant structures. These studies further illustrate the influence of canopy structure on airflow distribution and droplet deposition.

Optical porosity is an important indicator for quantitatively measuring canopy structure parameters. It is defined as the ratio of leaf gap area on a projection plane to the contour area of the canopy leaves (Loeffler et al., 1992; Zhu et al., 2003). To accurately describe and calculate porosity, researchers have conducted extensive studies using hemispherical photography (Qu et al., 2016), laser point clouds (Escolà et al., 2017), hyperspectral or thermal infrared techniques (Neinavaz et al., 2016) and physical and mathematical models (Giusti and Marsili-Libelli, 2006). However, these sensor-based calculation methods are costly and can only calculate the static porosity of canopy. In addition, the spatial distribution of leaves in canopy cannot be fully captured due to high canopy leaf density and heavy shading, especially in the late stages of crop growth. In fact, crops cultivated in the field have traceable patterns in growth and spatial distribution of leaves (Guo et al., 2009). According to these morphological characteristics (Liu et al. (2020, 2021a) specially studied the canopy porosity during spraying, proposed a 3D model to calculate the changes of canopy porosity, and realized the rapid prediction of crop canopy porosity. However, there is a strong interaction between leaves and airflow in the actual process of air-assisted spraying. The assisted airflow can not only move and deform the leaves, thus widening the droplet transport channel, but also improve the droplet transfer speed. It can effectively improve droplet deposition in the plant canopy and reduce drift (Panneton and Piché, 2005; Tang et al., 2021). Existing porosity calculation methods are difficult to express this physical process.

At present, the interaction between airflow and canopy is not clear. It is reflected in the following two aspects: one is how the airflow changes the deformation of the leaves, and the other is how the leaves affect the distribution of the airflow field. However, current research mainly calculates porosity based on the static structural characteristics of the canopy, and porosity is constantly changing in the actual spraying process. There is a lack of research on the quantitative description of porosity under assisted airflow, especially the dynamic changes in porosity. In our previous research, we studied a numerical simulation method for the fluid-structure interaction of leaves and spray airflow (Cui et al., 2023). In this study, we attempt to introduce the entire plant structure into numerical simulation and to calculate the real-time dynamic porosity. The relating between assisted airflow and canopy structural parameters is analyzed, and a new method for predicting and simulating canopy dynamic porosity is proposed.

## 2 Materials and methods

### 2.1 3D virtual cotton plant model and artificial cotton

**Figure 1:**
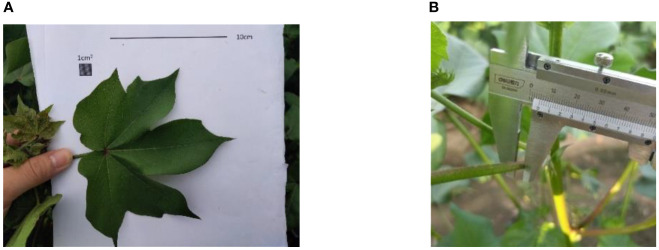
Measurement of Cotton Phenotypic Parameters. **(A)** leaf, **(B)** petiole.

In this paper, in order to build a 3D virtual plant model with reasonable simplification of the canopy, the main stems, fruiting branches, petioles, and leaves are mainly considered. According to previous studies (Liu et al., 2021a; Cui et al., 2022) and cotton field measurement data (**Figure 1**), the structural parameters of cotton, such as plant height, petiole length and diameter, and leaf shape are determined. The main stem nodes of cotton are divided into 20 segments. The 5-20 nodes are set as fruiting branch growth positions. The multiaxial branching of fruiting branches is simplified to straight branches, and the distance between nodes on the one fruiting branch is the same. The ratio of ovate, 3-lobed and 5-lobed leaves to the total number of leaves in a single cotton plant is 20%, 55% and 25%, respectively. Based on the collected phenotype data, the windward areas of ovate, 3-lobed and 5-lobed leaves are determined to be 4206.25 mm^2^, 7859.10 mm^2^ and 9823.00 mm^2^, respectively.

**Figure 2:**
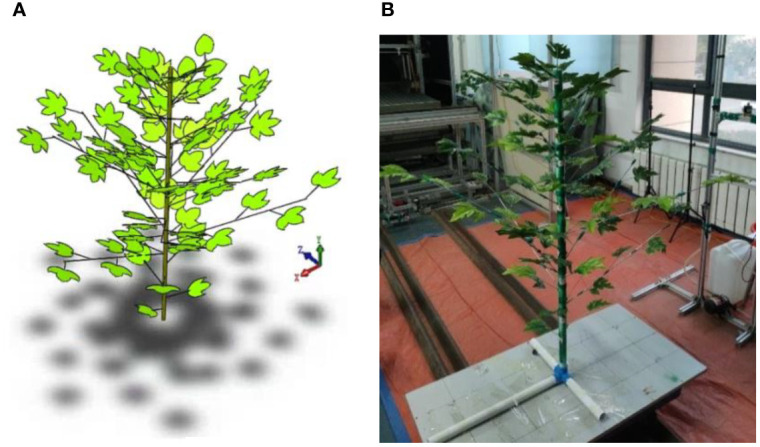
3D virtual cotton plant **(A)** and artificial cotton plant **(B)**.

The 3D phenotypic plant models are constructed in SolidWorks (2019, Dassault Systemes, FR) software. The cotton plant has a height of 130cm and is stratified into three layers (i.e., 0-50 cm along the main stem height direction as the lower layer, 50-90 cm as the middle layer, and 90-130 cm as the upper layer). Based on the morphological characteristics and growth pattern of cotton plants, a 3D virtual plant is constructed as shown in **Figure 2A**.

An artificial plant is built according to the morphological characteristics of the 3D virtual plant. The material of artificial cotton leaves is selected from cloth material based on the previous experiments of leaf deformation and spray retention (Liu et al., 2021a; Liu et al., 2021b). The main stem is made of PVC plastic pipe. Wire of suitable stiffness is used for the leaf stem and fruiting branch materials. The fruiting branches and leaves are placed on the pre-drilled holes in the main stem. The single artificial cotton plant is shown in **Figure 2B**.

### 2.2 Numerical approach

#### 2.2.1 Lattice Boltzmann model

*Lattice Boltzmann* (*LB*) model is suitable for solving many complex scientific problems. In particular, it does not require tracing the interface between different phases when dealing with multiphase and multi-component flows. In addition, it has been proven to have reliable accuracy in dealing with problems on microscopic and macroscopic scales (Ran and Xu, 2009; Men et al., 2017; Cui et al., 2023).

The important basis of the *LB* model is the theory of molecular motion. And *LB* model has the following assumptions.

a. The velocity component of each moving molecule is calculated without considering the influence of adjacent molecules.
b. The collision between two molecules is only considered.
c. The trajectory of each moving molecule is calculated without considering environmental factors.

Based on the above assumptions, the equation of molecular motion calculation function *f* is obtained. The independent variables of the equation are spatial velocity position vector, molecular velocity vector and time. The assumption on the molecular collision term can be simplified to a single relaxation time Bhatnagar Gross Krook (BGK) collision operator (Aidun and Clausen, 2010; Zhang et al., 2020). This simplification can effectively reduce the computational power. The simplified equation is the Boltzmann-BGK equation, expressed as (Aidun and Clausen, 2010):


(1) $${\text{f}}_{\text{α}}\left(r+{\text{K}}_{\text{α}}{\text{δ}}_{\text{t}},\;t+\delta_{\text{t}}\right)-{\text{f}}_{\text{α}}\left(\text{r},\text{t}\right)=-\frac{1}{\text{τ}}\left({\text{f}}_{\text{α}}\left(\text{r},\text{t}\right)-f_{\alpha }^{\text{eq}}\left(\text{r},\text{t}\right)\right)+{\text{δ}}_{\text{t}}{\text{F}}_{\text{α}}\left(\text{r},\text{t}\right)$$

Where, *r* is the position vector; *t* is the time, s; $f_{\alpha }$ is a discrete velocity distribution function; $K_{\alpha }$ is discrete particle velocity vector; ${\text{δ}}_{t}$ is the time step; τ is the dimensionless relaxation time, $\text{τ}=\tau_{0}/\delta_{t}$ ; $f_{\alpha }^{eq}$ is a local equilibrium distribution function; $F_{\alpha }$ is the external force term.

**Figure 3:**
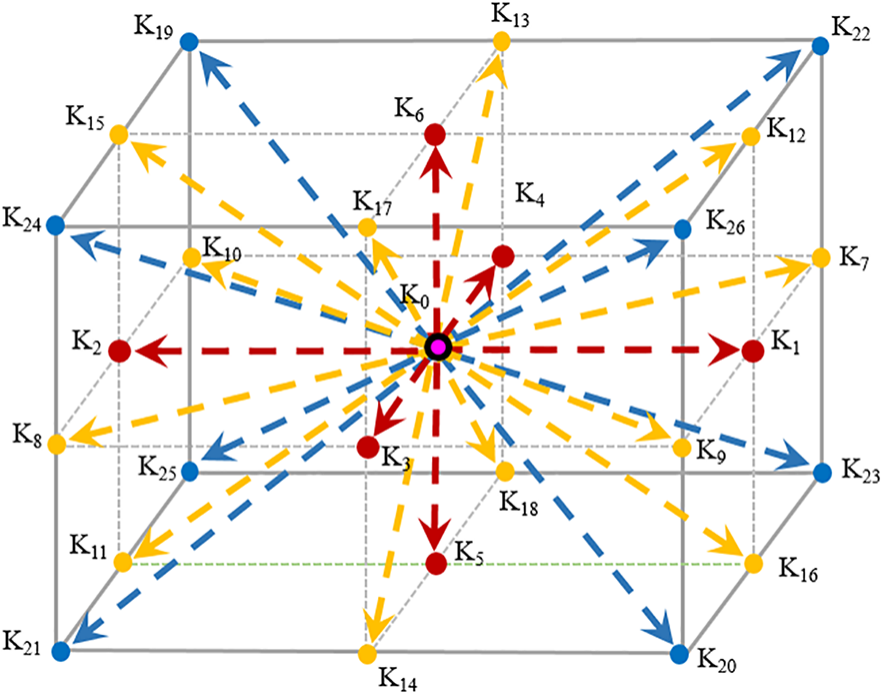
Schematic diagram of the *LB model* of D_3_Q_27_.

Through the discrete process, particles may move and collide, meaning that particles can move from one node to another in adjacent time steps while colliding with other adjacent particles. In addition, the *LB* method can calculate the macroscopic characteristics of the fluid through the statistical analysis of particles in the computational domain, and establish the relating between microscopic particles and macroscopic phenomena. The model *D*_*n*_ *Q*_*m*_ can be expressed as *n* dimensions and *m* discrete velocities. In this study, the octree lattice structure of D_3_ *Q*_27_ is used, as shown in **Figure 3**.

#### 2.2.2 Turbulence model

Large Eddy Simulation (LES) is used to model the turbulence distribution. This approach introduces an additional viscosity, called turbulent eddy viscosity to model the sub-grid turbulence (Weickert et al., 2010). The LES scheme adopts a wall-adapting local eddy viscosity model, which provides a consistent local eddy-viscosity and near-wall behavior (Ducros et al., 1998; Men et al., 2017; Zhang et al., 2020). The specific formulas are as follows:


(2) $${\text{U}}_{\text{t}}={\left({\text{B}}_{\text{w}}\text{Δ}\right)}^{2}\frac{{\left({\text{Q}}_{\text{αβ}}^{\text{d}}{\text{Q}}_{\text{αβ}}^{\text{d}}\right)}^{3/2}}{{\left({\text{Q}}_{\alpha \beta }{\text{Q}}_{\text{αβ}}\right)}^{5/2}+{\left({\text{Q}}_{\text{αβ}}^{\text{d}}{\text{Q}}_{\text{αβ}}^{\text{d}}\right)}^{5/4}}$$

(3) $${\text{Q}}_{\text{αβ}}=\frac{{\text{ɡ}}_{\text{αβ}}+{\text{ɡ}}_{\text{βα}}}{2}$$

(4) $${\text{Q}}_{\text{αβ}}^{\text{d}}=\frac{1}{2}\left({\text{ɡ}}_{\text{αβ}}^{2}+{\text{ɡ}}_{\text{βα}}^{2}\right)-\frac{1}{3}{\text{φ}}_{\text{αβ}}{\text{ɡ}}_{\text{rr}}^{2}$$

(5) $${\text{ɡ}}_{\text{αβ}}=\frac{\partial_{{\text{μ}}_{\text{α}}}}{\partial_{{\text{x}}_{\text{β}}}}$$

Where, *U_t_*, $B_{w}\Delta$ , Δ, and $\varphi_{\alpha \beta }$ are turbulent eddy viscosity, filter scale, unit grid scale, and Kronecker symbol, respectively. *Q*_αβ_ and $Q_{\alpha \beta }^{d}$ are the resolving scale strain rate tensors; $B_{w}$ (0.325) is a constant. ${ɡ}_{\alpha \beta }$ ${ɡ}_{\beta \alpha }$ and ${ɡ}_{rr}\;$ are the components of the strain rate tensor obtained from the second-order moment via the *LB* model. In the above equations, the subscripts *α*, *β* and *r* denote directions in space. The *µ* and *x* are velocity at a given distance from the wall and local flow direction tangential to the wall.

#### 2.2.3 Boundary conditions and computational domain

##### 2.2.3.1 Solid domain

In the fluid-structure coupling collaborative simulation, the setting size of stem and leaf parameters and the shape quality of mesh subdivision will have a significant impact on leaf deformation. The data interaction between the solid domain and the fluid domain should be completed by setting appropriate boundary conditions. It is necessary to establish the coupling environment required for collaborative simulation to ensure the accuracy of the simulation. The elements are divided in *Finite Element* (*FE*) solver, and the stem and leaf parameters and boundary conditions are set. Through two-way fluid-structure coupling, the change trend and deformation amount of leaves can be calculated, and then the dynamic porosity of leaves after deformation can be obtained.

**Figure 4:**
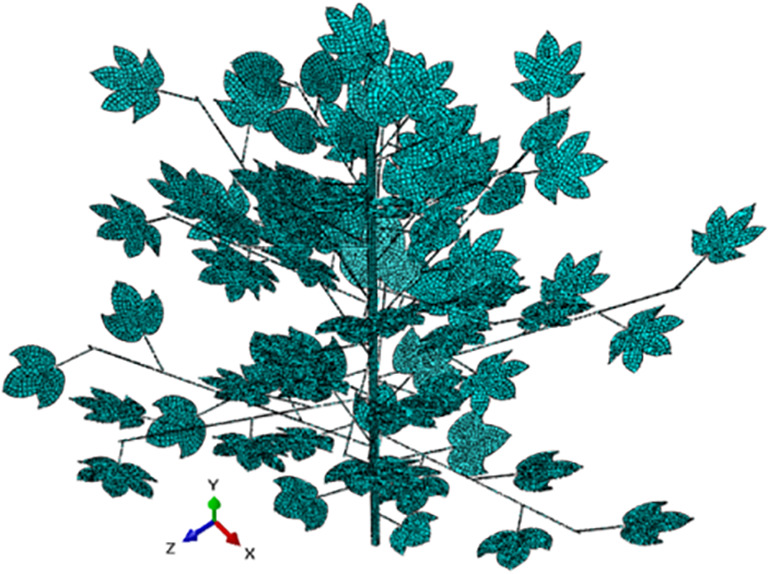
Schematic diagram of elements distribution in the structural explicit *FE* solver.

The accurate and reasonable segmentation of *FE* mesh is the basis of *FE* solver analysis. High-quality mesh can not only ensure reasonable analysis results, but also shorten the simulation time. Therefore, selecting a suitable element segmentation method is particularly important. The numerical integration method of *FE* solver adopts Gaussian numerical integration, and the integration points of different element shapes are different. The C3D8R element of linear hexahedron reduction integral is used for the element subdivision of stems, leaves and petioles, which is used as a three-dimensional eight-node linear solid element, namely hexahedron element. Each node has six degrees of freedom and can bend in any direction. The combined element subdivision of cotton plant is shown in **Figure 4**. The number of elements is 16966, and the number of nodes is 41981 and the element size is 9mm.

In the fluid-structure coupling analysis of assisted airflow and leaves, it is necessary to set the calculation attributes of the *FE* solver for the solid domain of stems and leaves.

(1) Material properties

The bending of stems and leaves affected by the assisted airflow is an elastic deformation phenomenon, so the elastic material parameters are used to simulate the stems and leaves. According to previous studies (Ma and Li, 2014; Liu et al., 2021a; Cui et al., 2023), the elastic modulus of cotton leaves is set to 46.5Mpa, the Poisson’s ratio of leaves is 0.32, and the density of leaves is 700 kg·m^-3^.

(2) Analysis step settings

In *FE* solver, the dynamic display analysis is set up. Since the spraying device drives the air curtain and nozzle to move in the actual spraying process. The initial value is verified according to the maximum time step of the fluid domain pilot site. The solid domain analysis step is set to 1s, and the field output adjusts 1s to 200 uniform time intervals, that is, there are 200 imaging effects within 1s.

(3) Boundary condition

According to the distribution and connection of stems and leaves, the location of stem is defined as a completely fixed constraint in the boundary conditions setting, namely 0 degrees of freedom. In the simulation process, it only bears the influence of airflow and no external force, so it is only necessary to set the corresponding contact attributes.

##### 2.2.3.2 Fluid domain

**Figure 5:**
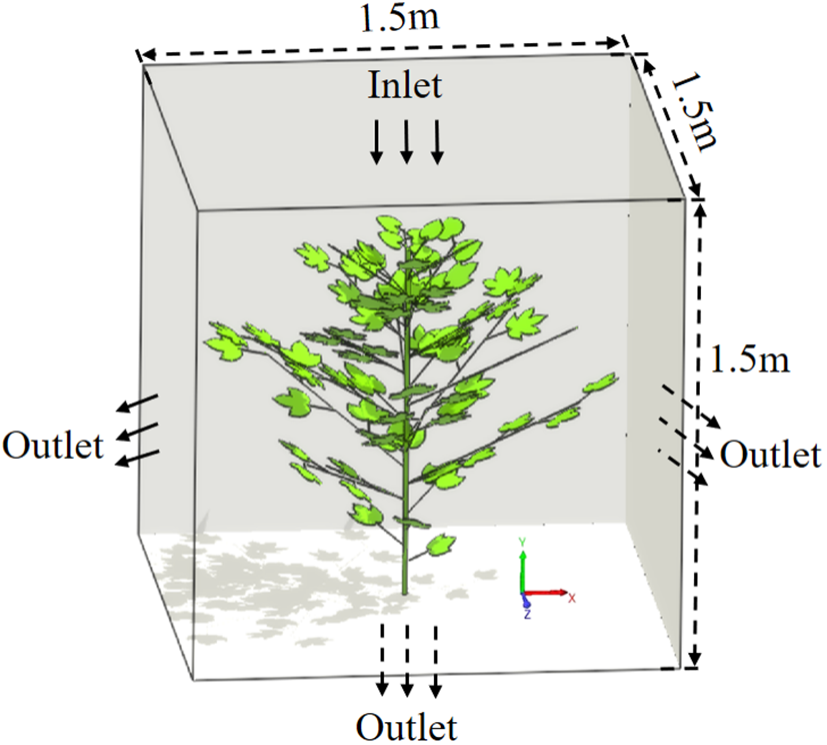
Schematic diagram of the distribution of cotton plant and air domain locations.

The fluid domain model is the air domain model. The plant should be placed in the air domain. The air domain model is built in 3D modeling software. The size is 1500 mm long, 1500 mm wide and 1500 mm high. This model is saved in a file format that can interface with the *LB* solver, and is named as ‘air.stl’. The imported air fluid domain uses meshless modeling. The coordinate position of the fluid domain is adjusted to ensure that the branches and leaves are all in the flow field analysis domain. The location distribution of solid domain and fluid domain models is shown in **Figure 5**. The gravity acceleration applied to the fluid is set to -9.81m·s^-2^. The default material ‘Material 1’ is used in the fluid domain, and its relevant parameters are set as follows: the molecular weight of air is 28.996 g·mol^-1^, the density of air is 1.225 kg·m^-3^, and the operating temperature is 289.35 K (16.2°C). The gas flowing at low speed is a Newtonian fluid, so the dynamic viscosity is set to 1.7894e-05 Pa ·s. In air-assisted spraying, the downward airflow can open the upper branches and leaves, which plays an important role in increasing the amount of droplets deposition in the lower and middle layers in dense canopy (Zhang et al., 2022; Zhu et al., 2022). In this study, we mainly consider the effect of downward airflow on porosity changes.

#### 2.2.4 Fluid-structure coupling module

**Figure 6:**
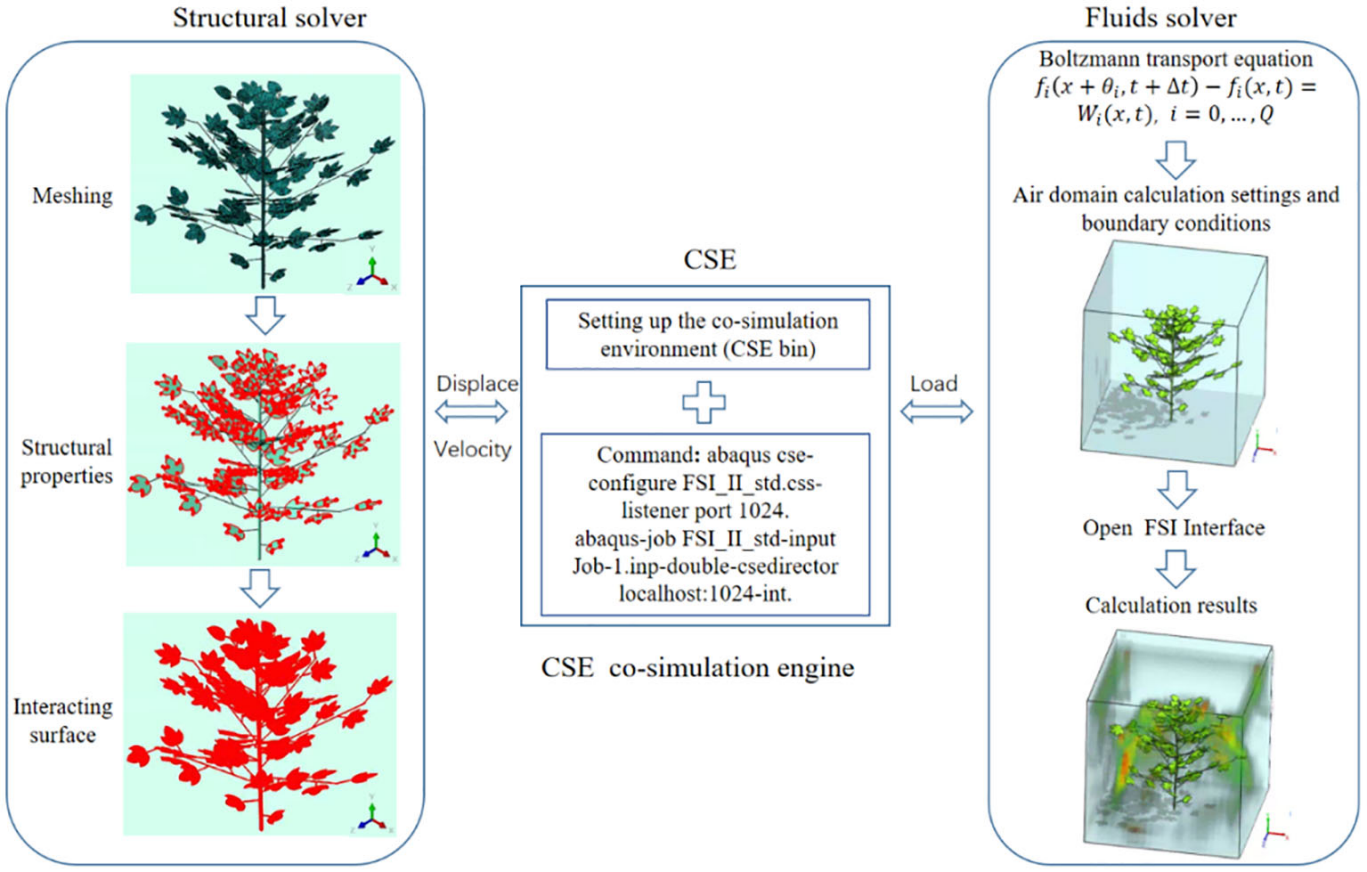
Flow chart of the fluid-structure coupling approach.

In *FE* solver, the contact surface with the fluid domain is set as the fluid-structure co-simulation boundary, as shown in **Figure 6**. After completing the above steps, set the calculation file in the Job module, name it *Job*-1, and export the.*inp* file. The recognition subroutine of fluid-structure coupling interface is set up in the.*inp* file. The flowchart of the fluid-structure coupling approach is shown in **Figure 6**.

#### 2.2.5 Validation of numerical simulation

**Figure 7:**
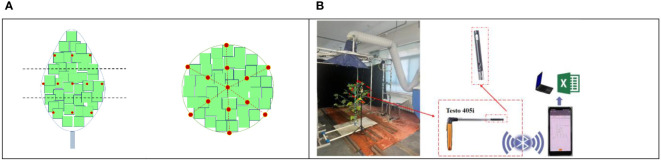
Distribution of sampling points **(A)** and indoor airflow measurement **(B)**.

In order to make the experimental conditions controllable and avoid interference of uncontrollable factors in the natural environment, in this paper, we construct an artificial cotton plant based on the 3D virtual cotton plant (Dekeyser et al., 2014; Cui et al., 2022). To validate the numerical simulation, the differences between simulated and experimental values at the same position in the canopy are compared. The positions of the sampling points are as shown in **Figures 7A, B**. In **Figure 7B**, the centrifuge (4-72-6A, FOSHAN CITY NANHAI POPULA FAN CO., LTD, China) is the wind source and the variable frequency speed controller is used to regulate the speed. The air velocity at the sampling points was measured separately by a hot-wire anemometer (Testo 405i, Titisee-Neustadt, Germany). The data measured by the hot-wire anemometer are exported to the computer terminal via Bluetooth.

In simulation, the airflow velocity at the sampling point is output through the detection line. The normalized mean absolute error (NMAE) between the measured and the simulated values of the upper, middle, and lower layers of the canopy were compared. The total difference was analyzed by fitting a linear equation.

### 2.3 Dynamic changes in canopy porosity based on image processing

Optical porosity is defined as the ratio of canopy leaf void area to contour plane area on a projection plane (Loeffler et al., 1992; Zhu et al., 2003). It is related to plant density, structure and environmental conditions, and is a structural parameter closely related to flow and resistance characteristics near plants. In pesticide spraying, leaves are the main organ of droplet deposition. In addition to the size and position of the leaf, the leaf will bend and deform due to airflow disturbance, and the optical porosity will change accordingly.

**Figure 8:**
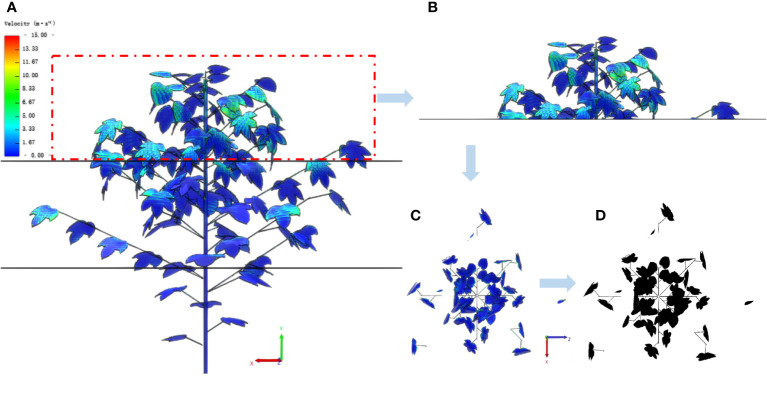
Calculation process of canopy stratified porosity. **(A)** The front view of a stratified cotton plant. **(B)** The front view of the upper layer. **(C)** The top view of the upper layer. **(D)** The image processing result of the top view.

Based on the 3D model of the plant and the fluid-structure coupling process, we propose a method to calculate the target canopy porosity by layering and zoning based on image processing. This method can obtain canopy dynamics images during the interaction between canopy and airflow through CFD post-processing, and then use image processing to calculate porosity at any canopy height. The specific process is as follows:

(1) Image acquisition. Using the layer plane as the reference plane, images of different canopy positions of three-dimensional cotton plant targets along the reference plane are captured and stored in *LB* solver (**Figures 8A–C**).
(2) Image processing. Canopy projection images are processed by image denoising, gray scale processing, thresholding and binarization (**Figure 8D**).
(3) Determination of canopy outer edge. Determine the canopy projection along the outermost leaf edge of the canopy and calculate the projection area (S_i_).
(4) Calculation of windward area. The pixel value occupied by the plant projection is counted, and according to the image and 3D plant leaf scale, the leaf windward area (A_i_) at different canopy positions of the plant is calculated.
(5) Calculation of stratified porosity. The porosity of each layer can be calculated using the following formula: ${\text{P}}_{\text{i}}=\frac{{\text{A}}_{\text{i}}}{\text{Si}}$ .

## 3 Results and discussions

### 3.1 Simulation validation results

**Figure 9:**
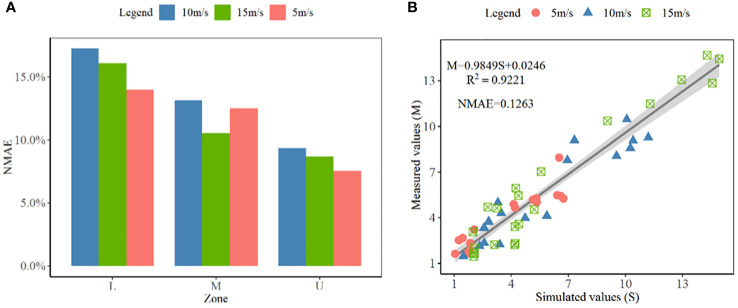
The comparative analysis between the measured values and the simulated values. **(A)** is the NMAE between simulated and measured values; **(B)** is the linear fitting analysis between simulated and measured values.

In this study, in order to verify the reliability of the numerical simulation results, several sampling points are set up in the upper, middle, and lower layers of the artificial cotton planting target area. The initial airflow velocity values are 5 m·s^-1^, 10 m·s^-1^ and 15 m·s^-1^, respectively. The results are shown in **Figure 9A**. Under the initial air velocity of 5 m·s^-1^, the normalized mean absolute error (NMAE) between the simulated value and the measured value of the upper, middle and lower parts of the canopy are 7.54%, 12.51% and 13.99%, respectively. Under the speed of 10 m·s^-1^, the NMAE are 9.36%, 13.14% and 20.72%, respectively. Under the speed of 15 m·s^-1^, the NMAE are 8.69%, 10.54% and 16.08%, respectively. The results show that the simulated airflow velocity distribution can reflect the attenuation of airflow velocity in the canopy. To further verify the accuracy of the simulation results, linear fitting analysis is performed on all simulated values (S) and measured values (M), as shown in **Figure 9B**. The expression of the linear equation is M=0.9849S+0.0246, and the coefficient of determination (R^2^) is 0.9221. The fitting results indicate that the fluid-structure coupling method can effectively reflect the dynamic changes of canopy porosity.

### 3.2 Leaf deformation and porosity changes in the canopy at different airflow velocities

**Figure 10:**
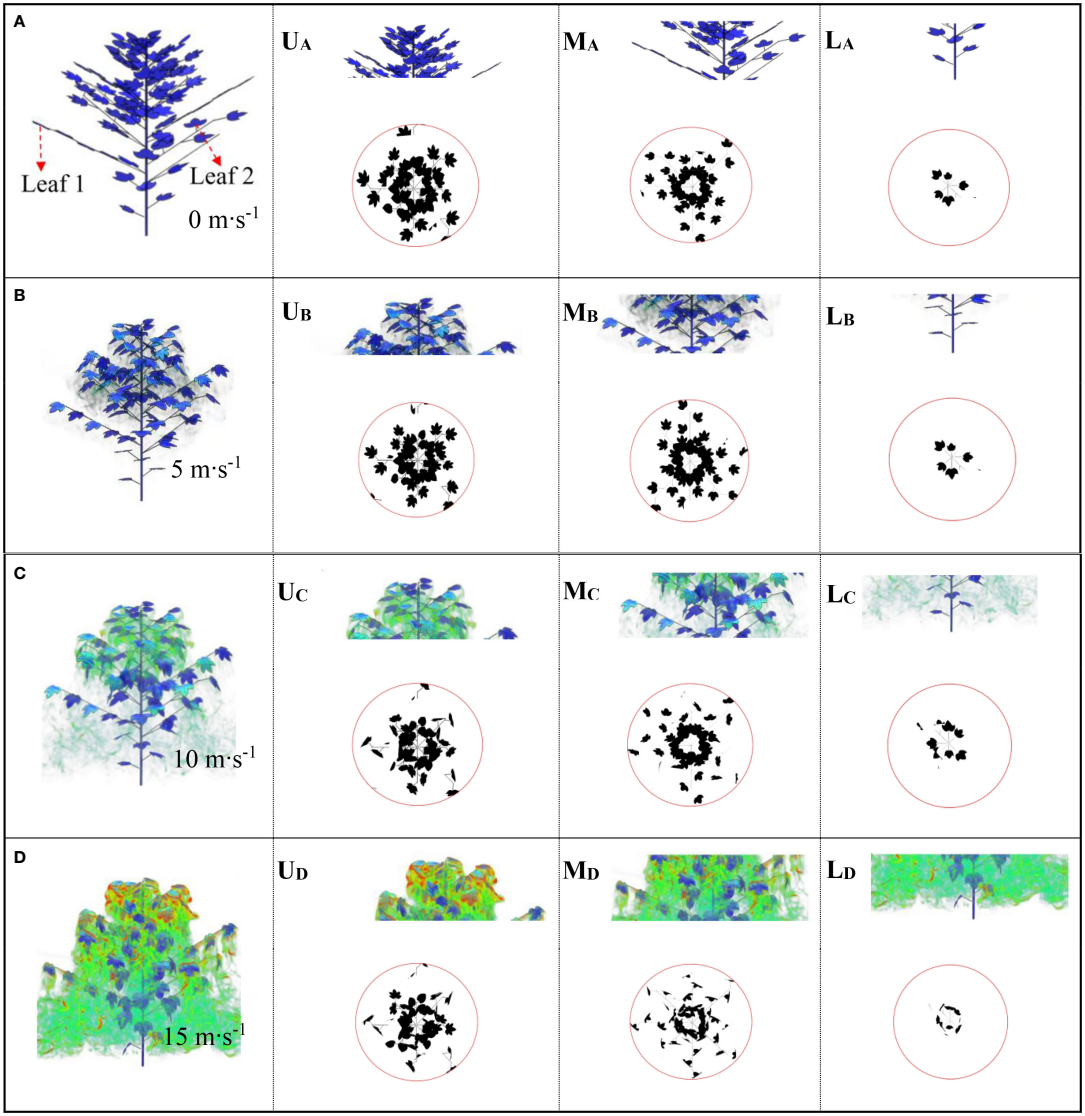
Airflow distribution at different initial wind speeds at 0.1s. **(A)** is 0 m·s-1; **(B)** is 5 m·s-1; **(C)** is 10 m·s-1; **(D)** is 15 m·s-1'. U, M and L represent the upper, middle, and lower layers of the canopy, respectively.

**Figure 10** shows the leaf deformation and porosity changes within the canopy under the influence of different airflow velocities at 0.1s. Under 0 m·s^-1^, 5 m·s^-1^, 10 m·s^-1^ and 15 m·s^-1^, the upper, middle and lower canopy leaves have different degrees of deformation, and the airflow velocity and vortex distribution changes are different.

In the absence of wind (0 m·s^-1^), we export images at different heights of the canopy and calculated the porosity of the upper, middle, and lower layers as 49.95%, 59.85% and 49.16%, respectively (**Figure 10A**). When the airflow velocity reaches 5 m·s^-1^, we find that the leaves at different heights showed slight twisting or bending deformation (**Figure 10B**). Compared with the velocity of 0 m·s^-1^, the porosity at the same canopy position is 52.27%, 52.23% and 49.74%, respectively. **Figure 10C** shows the changes in leaves within the canopy at the same time when the airflow speed increases to 10 m·s^-1^. The porosity at different canopy heights is 66.83%, 64.16% and 38.71%, respectively. As the flow rate increases, the plant shrinks its shape, rolls up its leaves and bends downstream, resulting in a decrease in its cross-sectional area and an increase in its fluidization degree. **Figure 10D** shows the changes in leaves within the canopy at the same time when the airflow speed increases to 15 m·s^-1^, with the porosity of each layer being 68.07%, 73% and 62.35%, respectively. As the wind load increases, the leaves sway more and become more random. Compared with the initial state of 5 m·s^-1^, the change ratio has increased by 36.28%, 21.97% and 26.83%, respectively. From the perspective of leaf deformation, when subjected to increased airflow load, the distribution of canopy branches and leaves will be reconfigured to reduce wind force and absorb momentum in the airflow, which is a reconstruction phenomenon (Ennos et al., 2000; Vollsinger et al., 2005; Kane et al., 2008; Miri et al., 2018). This behavior is often observed in plants with flat and thin leaves (Gillies et al., 2002). At higher wind speeds, plant resistance decreases and leaf deformation increases. However, in actual spraying operations, the distribution of vortices around the canopy changes greatly, which increases the drift of droplets in the atmosphere.

**Figure 11:**
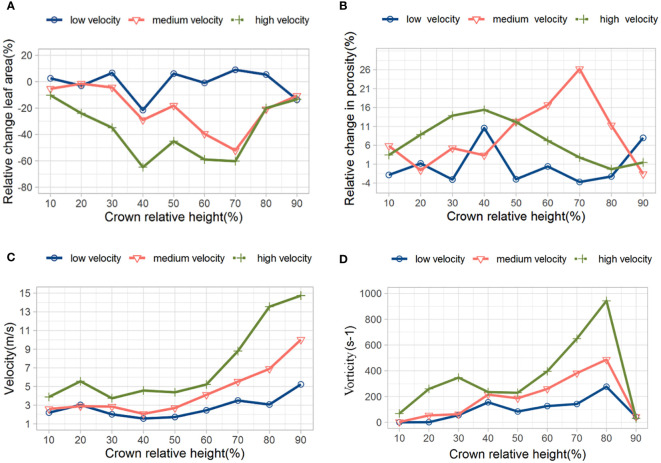
Changes in windward area **(A)**, porosity **(B)**, velocity **(C)**, and vorticity **(D)** at different CRHs.

We derive the windward area and porosity at different canopy relative heights (CRHs) (10%, 20%,…, 100%) to further describe the changes in leaf deformation and porosity along the depth of the canopy. It is assumed that the initial porosity is defined as the porosity at different positions of the canopy under the condition of no wind (0 m·s^-1^). The dynamic variation of porosity is defined as the change in porosity relative to the initial porosity at different relative heights of the canopy under wind conditions (5 m·s^-1^, 10 m·s^-1^, 15 m·s^-1^). **Figure 11A** shows the changes in windward leaf area at different relative heights of the canopy under different initial velocities. From **Figure 11A**, it can be seen that the changes in leaf area at the bottom (CRH=10%) and top (CRH=90%) of the canopy are relatively small. While the changes in windward area in the middle of the canopy (CRH=20% -80%) are relatively large. Under low wind speed (5 m·s^-1^), the maximum change in the windward area of leaves at different CRHs is 20%. As the airflow speed increases, the changes in the windward area of leaves within the canopy under 10 m·s^-1^ and 15 m·s^-1^ are significantly larger than those under low wind speed conditions. These results indicate that low wind speeds cause slight vibration of the leaves, while high wind speeds cause significant bending or deformation of the leaves. **Figure 11B** shows the dynamic changes in porosity at each relative height of the canopy. Under the three wind speed conditions of low (5 m·s^-1^), medium (10 m·s^-1^), and high (15 m·s^- 1^), the relative heights of the canopy with the largest changes in porosity are 40%, 70%, and 40%, respectively, and the maximum changes in porosity are 11%, 26%, and 16%, respectively. The maximum change in porosity occurs in the middle of the canopy because the interaction between airflow and leaves becomes stronger in areas with denser leaves. Comparing **Figures 11A, B**, we find that there is consistency between the position with the largest change in leaf windward area and the position with the largest change in porosity. The specific modification form is as follows: 'the position with the largest change in porosity. **Figures 11C–D** and show the changes in velocity and vorticity at different relative heights of the canopy. As the depth of the canopy increases, the airflow velocity continues to decay. At CRH=80%, a significant change in vorticity occurred, which is related to the obstruction of airflow by the upper branches and leaves of the canopy.

### 3.3 Dynamic changes in porosity at different times

**Figure 12:**
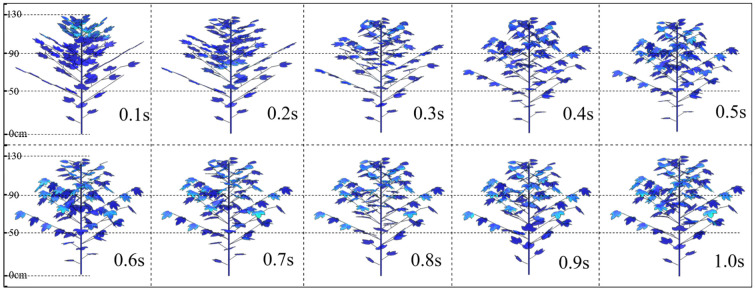
Changes in canopy leaf morphology at different times under 5 m·s^-1^.

**Figure 12** shows the dynamic changes in porosity at different times (0-1s) in the upper, middle and lower layers of the canopy at a wind speed of 5 m·s^-1^ (light breeze). At 0.1s, the upper leaves of the canopy begin to deform first due to the influence of the upper airflow, and the upper layer undergoes changes earlier than the middle and lower layers. From 0.2s to 0.5s, the leaves within the canopy continuously bend downward and deform. From the perspective of the curvature amplitude of most leaves in the canopy, the curvature degree of the canopy leaves is the highest at 0.6s. After 0.7s, the leaves gradually rebound. Due to the obstruction of airflow by the upper and middle branches and leaves within the canopy, the intensity of airflow decreases greatly when it reaches the lower part. The airflow ultimately causes only slight deformation of the leaves in the lowest layer, indicating that under the action of a small airflow (light breeze), the deformation amplitude of the canopy leaves is small and they sway slightly up and down.

**Figure 13:**
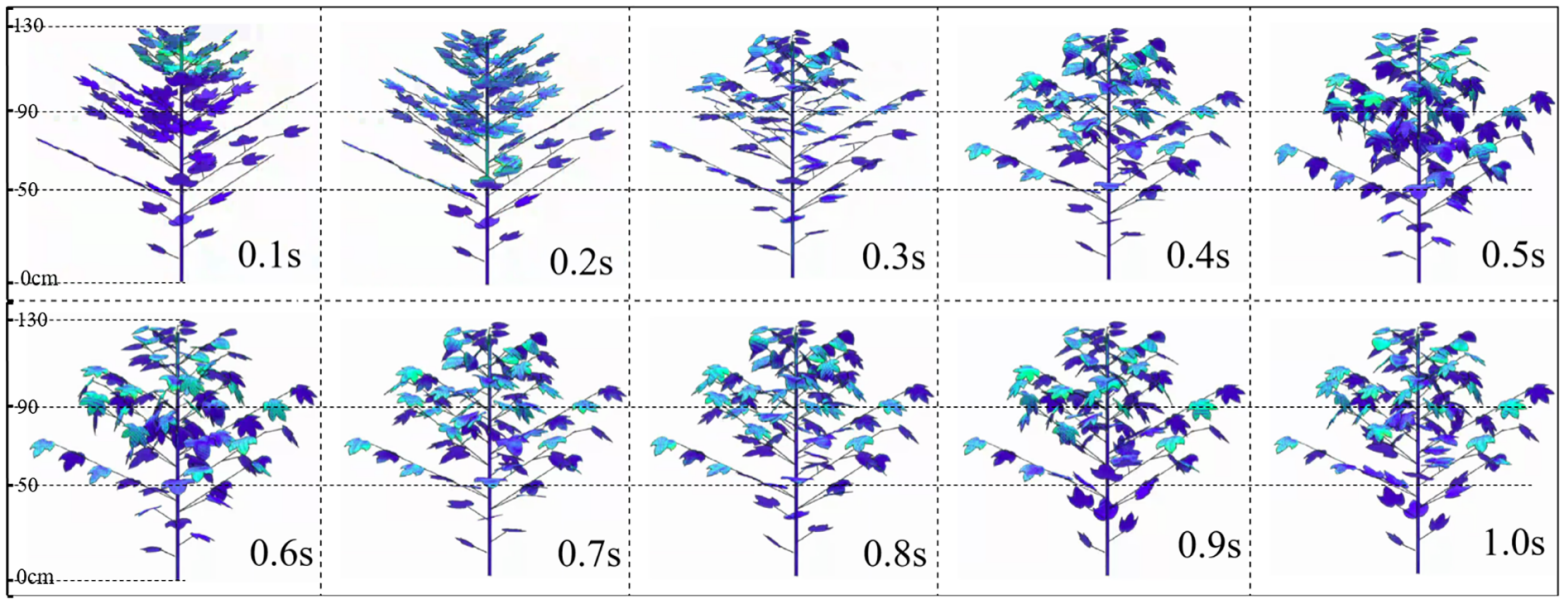
Changes in canopy leaf morphology at different times under 10 m·s^-1^.

**Figure 13** shows the deformation of internal branches and leaves in the canopy under airflow disturbance at different times when the assisted airflow velocity is 10 m·s^-1^ (moderate wind). At 0.1s, the airflow has just reached the top of the canopy. At 0.2s to 0.4s, the canopy leaves begin to gradually bend downward. Compared to the initial wind speed of 5 m·s^-1^, the leaf reaches its maximum deformation at 0.5s. At this moment, the maximum porosity of the entire canopy indicates that the moment when the wind speed increases and the maximum leaf deformation occurs is relatively late. After 0.6s, the deformation amplitude of most leaves in the canopy shows a slight rebound. A small number of leaves have a large rebound amplitude and even show a reversal trend, especially the leaves at the bottom of the canopy have obvious deformation (0.9s to 1.0s). This may be caused by the vortices formed under the canopy. Due to the obstruction of the upper leaves, the assisted airflow under the canopy gradually decreases, and the intensity of the vortices on the back of the lower leaves is greater than that of the assisted airflow on the front. This leaf inversion is very important for the uniform deposition in the middle and lower parts of the canopy during spraying.

**Figure 14:**
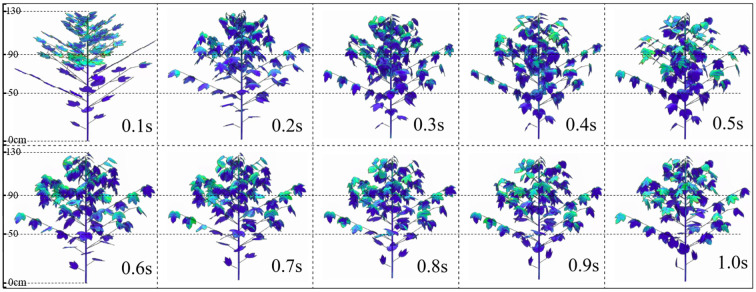
Changes in canopy leaf morphology at different times under 15 m·s^-1^.

**Figure 14** shows the deformation of internal branches and leaves in the canopy under airflow disturbance at different times when the assisted airflow velocity is 15 m·s^-1^ (high wind speed). At 0.2s, the airflow has reached the lower part of the canopy and caused deformation of the lowest leaves. As the airflow gradually enters the interior of the canopy, the leaf deformation amplitude reaches its maximum value at 0.4s, which is earlier than the maximum moment of leaf deformation under medium (10 m·s^-1^) and low (5 m·s^-1^) wind speeds, indicating that the leaves are susceptible to deformation under high wind speeds. After 0.5s, the leaves in the canopy become disorderd, floating up and down, and some of the leaves undergo reversals, especially for 0.9s and 1.0s. From the perspective of leaf deformation, the degree of leaf deformation will not increase as the airflow velocity continues to increase. However, a higher velocity can lead to an increase in airflow disturbance outside the canopy, which leads to the possibility of spray droplets drift.

## 4 Conclusions

In this study, a new method based on CFD simulation and image processing is proposed to calculate the dynamic changes in porosity caused by leaf deformation under assisted airflow. A two-way fluid-structure interaction model is developed based on LB solver and FE solver. The model achieves a quantitative and intuitive analysis of the deformation of leaves in the canopy under the action of assisted airflow. The fluid-solid interaction model is validated by indoor experiments. The results of CFD post-processing are analyzed using image processing algorithms, and the stratified dynamic porosity in the canopy is calculated at different velocities and different times. This study provides an idea to clarify the dynamic changes of porosity in canopy during air-assisted spraying and to analyze the mechanism of increasing droplet deposition in canopy by assisted airflow.

Compared with previous static porosity studies, this study can better reveal the dynamic interaction phenomenon between crop leaves and airflow. However, the developed CFD model still needs further improvement. In order to accurately predict the dynamic porosity of the canopy, 3D leaf modeling should consider more details. In addition, the effect of different directional assisted airflow on the canopy structure should be considered.

## Data availability statement

The raw data supporting the conclusions of this article will be made available by the authors, without undue reservation.

## Author contributions

HC: Conceptualization, Methodology, Investigation, Validation, Data curation, Software, Writing-original draft, Writing-review & editing. CW: Conceptualization, Methodology, Data curation, Writing-review & editing, Visualization, Investigation. XL: Project administration, Funding acquisition, Resources. JY: Supervision, Funding acquisition, Writing-Review & Editing. FL: Conceptualization, Methodology, Investigation, Data curation, Validation.

## References

[B1] Aidun C. K. Clausen J. R. (2010). Lattice-Boltzmann method for complex flows. Annu. Rev. fluid mechanics 42, 439–472. doi: 10.1016/j.soilbio.2017.12.011

[B2] Badules J. Vidal M. Boné A. Llop J. Salcedo R. Gil E.. (2018). Comparative study of CFD models of the air flow produced by an air-assisted sprayer adapted to the crop geometry. Comput. Electron. Agric. 149, 166–174. doi: 10.1016/j.compag.2017.09.026

[B3] Chen Y. Ozkan H. E. Zhu H. Derksen R. C. Krause C. R. (2013a). Spray deposition inside tree canopies from a newly developed variable-rate air-assisted sprayer. Trans. ASABE 56 (6), 1263–1272. doi: 10.13031/trans.56.9839

[B4] Chen Y. Zhu H. Ozkan H. E. Derksen R. C. Krause C. R. (2013b). Spray drift and off-target loss reductions with a precision air-assisted sprayer. Trans. ASABE 56 (6), 1273–1281. doi: 10.13031/trans.56.10173

[B5] Cross J. V. Walklate P. J. Murray R. A. Richardson G. M. (2001a). Spray deposits and losses in different sized apple trees from an axial fan orchard sprayer: 1. Effects of spray liquid flow rate. Crop Prot. 20 (1), 13–30. doi: 10.1016/S0261-2194(00)00046-6

[B6] Cross J. V. Walklate P. J. Murray R. A. Richardson G. M. (2001b). Spray deposits and losses in different sized apple trees from an axial fan orchard sprayer: 2. Effects of spray quality. Crop Prot. 20 (4), 333–343. doi: 10.1016/S0261-2194(00)00163-0

[B7] Cui H. Wang C. Liu X. Yuan J. Liu Y. (2023). Dynamic simulation of fluid-structure interactions between leaves and airflow during air-assisted spraying: A case study of cotton. Comput. Electron. Agric. 209, 107817. doi: 10.1016/j.compag.2023.107817

[B8] Cui H. Wang C. Liu X. Yuan J. Liu Y. Song L. (2022). Cotton canopy airflow simulation and velocity attenuation model based upon 3D phenotype and stratified sub-regional porous medium. Comput. Electron. Agric. 201, 107282. doi: 10.1016/j.compag.2022.107282

[B9] Damalas C. A. Eleftherohorinos I. G. (2011). Pesticide exposure, safety issues, and risk assessment indicators. Int. J. Environ. Res. Public Health 8 (5), 1402–1419. doi: 10.3390/ijerph8051402 21655127 PMC3108117

[B10] Da Silva A. Sinfort C. Tinet C. Pierrat D. Huberson S. (2006). A Lagrangian model for spray behavior within vine canopies. J. aerosol Sci. 37 (5), 658–674. doi: 10.1016/j.jaerosci.2005.05.016

[B11] Dekeyser D. Foqué D. Duga A. T. Verboven P. Hendrickx N. Nuyttens D. (2014). Spray deposition assessment using different application techniques in artificial orchard trees. Crop Prot. 64, 187–197. doi: 10.1016/j.cropro.2014.06.008

[B12] Derksen R. C. Zhu H. Ozkan H. E. Hammond R. B. Dorrance A. E. Spongberg A. L. (2008). Determining the influence of spray quality, nozzle type, spray volume, and air-assisted application strategies on deposition of pesticides in soybean canopy. Trans. ASABE 51 (5), 1529–1537. doi: 10.13031/2013.25301

[B13] Dorr G. Hanan J. Adkins S. Hewitt A. O’Donnell C. Noller B. (2008). Spray deposition on plant surfaces: a modelling approach. Funct. Plant Biol. 35 (10), 988–996. doi: 10.1071/FP08056 32688848

[B14] Ducros F. Nicoud F. Poinsot T. (1998). Wall-adapting local eddy-viscosity models for simulations in complex geometries. Numerical Methods Fluid Dynamics VI, 293–299 Available at:. https://www.imag.umontpellier.fr/~nicoud/PDF/ICFD_WALE.pdf.

[B15] Duga A. T. Ruysen K. Dekeyser D. Nuyttens D. Bylemans D. Nicolai B. M.. (2015). Spray deposition profiles in pome fruit trees: Effects of sprayer design, training system and tree canopy characteristics. Crop Prot. 67, 200–213. doi: 10.1016/j.cropro.2014.10.016

[B16] Endalew A. M. Debaer C. Rutten N. Vercammen J. Delele M. A. Ramon H.. (2010). A new integrated CFD modelling approach towards air-assisted orchard spraying. Part I. Model development and effect of wind speed and direction on sprayer airflow. Comput. Electron. Agric. 71 (2), 128–136. doi: 10.1016/j.compag.2009.11.005

[B17] Endalew A. M. Hertog M. Delele M. A. Baetens K. Persoons T. Baelmans M.. (2009). CFD modelling and wind tunnel validation of airflow through plant canopies using 3D canopy architecture. Int. J. Heat Fluid Flow 30 (2), 356–368. doi: 10.1016/j.ijheatfluidflow.2008.12.007

[B18] Ennos A. R. Spatz H. C. Speck T. (2000). The functional morphology of the petioles of the banana, Musa textilis. J. Exp. Bot. 51 (353), 2085–2093. doi: 10.1093/jexbot/51.353.2085 11141182

[B19] Escolà A. Martínez-Casasnovas J. A. Rufat J. Arnó J. Arbonés A. Sebé F.. (2017). Mobile terrestrial laser scanner applications in precision fruticulture/horticulture and tools to extract information from canopy point clouds. Precis. Agric. 18, 111–132. doi: 10.1007/s11119-016-9474-5

[B20] Gil Y. Sinfort C. (2005). Emission of pesticides to the air during sprayer application: A bibliographic review. Atmospheric Environ. 39 (28), 5183–5193. doi: 10.1016/j.atmosenv.2005.05.019

[B21] Gillies J. A. Nickling W. G. King J. (2002). Drag coefficient and plant form response to wind speed in three plant species: Burning Bush (Euonymus alatus), Colorado Blue Spruce (Picea pungens glauca.), and Fountain Grass (Pennisetum setaceum). J. Geophysical Research: Atmospheres 107 (D24), ACL–A10. doi: 10.1029/2001JD001259

[B22] Giusti E. Marsili-Libelli S. (2006). An integrated model for the Orbetello lagoon ecosystem. Ecol. Model. 196 (3-4), 379–394. doi: 10.1016/j.ecolmodel.2006.02.016

[B23] Grella M. Gioelli F. Marucco P. Zwertvaegher I. Mozzanini E. Mylonas N.. (2022). Field assessment of a pulse width modulation (PWM) spray system applying different spray volumes: duty cycle and forward speed effects on vines spray coverage. Precis. Agric. 23 (1), 219–252. doi: 10.1007/s11119-021-09835-6

[B24] Guo Y. Zhao C. Zhu Y. Li C. Sun H. Cao W. (2009). Morphogenesis model with relation to light and temperature condition for above-ground organs in cotton. Acta Agronomica Sin. 35 (11), 2101–2106. doi: 10.3724/SP.J.1006.2009.02101

[B25] Kane B. Pavlis M. Harris J. R. Seiler J. R. (2008). Crown reconfiguration and trunk stress in deciduous trees. Can. J. For. Res. 38 (6), 1275–1289. doi: 10.1139/X07-225

[B26] Khot L. R. Ehsani R. Albrigo G. Larbi P. A. Landers A. Campoy J.. (2012). Air-assisted sprayer adapted for precision horticulture: Spray patterns and deposition assessments in small-sized citrus canopies. Biosyst. Eng. 113 (1), 76–85. doi: 10.1016/j.biosystemseng.2012.06.008

[B27] Li X. Giles D. K. Niederholzer F. J. Andaloro J. T. Lang E. B. Watson L. J. (2021). Evaluation of an unmanned aerial vehicle as a new method of pesticide application for almond crop protection. Pest Manage. Sci. 77 (1), 527–537. doi: 10.1002/ps.6052 32816397

[B28] Liu X. Liu X. Li Y. Yuan J. Li H. (2021a). Predicting spray deposit distribution within a cotton plant canopy based on canopy stratification porosity and Gaussian process models. Biosyst. Eng. 204, 1–14. doi: 10.1016/j.biosystemseng.2020.12.018

[B29] Liu X. Liu X. Li Y. Yuan J. Song L. Li H.. (2020). Estimation model of canopy stratification porosity based on morphological characteristics: a case study of cotton. Biosyst. Eng. 193, 174–186. doi: 10.1016/j.biosystemseng.2020.02.018

[B30] Liu X. M. Song L. Q. Cui H. Y. Liu Y. C. Liu X. H. Wu M. Q. (2021b). Decoupling on influence of air droplets stress and canopy porosity change on deposition performance in air-assisted spray. Nongye Jixie Xuebao/Transactions Chin. Soc. Agric. Machinery 52 (8), 117–126+137. doi: 10.6041/j.issn.1000-1298.2021.08.011

[B31] Loeffler A. E. Gordon A. M. Gillespie T. J. (1992). Optical porosity and windspeed reduction by coniferous windbreaks in Southern Ontario. Agroforestry Syst. 17 (2), 119–133. doi: 10.1007/BF00053117

[B32] Ma L. F. Li L. Z. (2014). The topology optimization simulation study on the process of natural selection of leaf veins. Chin. J. Appl. Mech. 01), 132–136+12. doi: 10.11776/cjam.31.01.B027

[B33] Men Y. Lai Y. Dong S. Du X. Liu Y. (2017). Research on CO dispersion of a vehicular exhaust plume using Lattice Boltzmann Method and Large Eddy Simulation. Transportation Res. Part D: Transport Environ. 52, 202–214. doi: 10.1016/j.trd.2017.03.012

[B34] Miri A. Dragovich D. Dong Z. (2018). The response of live plants to airflow–Implication for reducing erosion. Aeolian Res. 33, 93–105. doi: 10.1016/j.aeolia.2018.06.002

[B35] Müller M. Rakocevic M. Caverzan A. Boller W. Chavarria G. (2018). Architectural characteristics and heliotropism may improve spray droplet deposition in the middle and low canopy layers in soybean. Crop Sci. 58 (5), 2029–2041. doi: 10.2135/cropsci2017.11.0653

[B36] Neinavaz E. Skidmore A. K. Darvishzadeh R. Groen T. A. (2016). Retrieval of leaf area index in different plant species using thermal hyperspectral data. ISPRS J. photogrammetry Remote Sens. 119, 390–401. doi: 10.1016/j.isprsjprs.2016.07.001

[B37] Olesen J. E. Jørgensen L. N. Petersen J. Mortensen J. V. (2003). Effects of rates and timing of nitrogen fertilizer on disease control by fungicides in winter wheat. 2. Crop growth and disease development. J. Agric. Sci. 140 (1), 15–29. doi: 10.1017/S0021859602002897

[B38] Panneton B. Piché M. (2005). Interaction between application volume, airflow, and spray quality in air-assisted spraying. Trans. ASAE 48 (1), 37–44. doi: 10.13031/2013.17938

[B39] Qiu W. Guo H. Cao Y. Li X. Wu J. Chen Y.. (2022). An electrical vortex air-assisted spraying system for improving droplet deposition on rice. Pest Manage. Sci. 78 (10), 4037–4047. doi: 10.1002/ps.7023 35638857

[B40] Qu Y. Meng J. Wan H. Li Y. (2016). Preliminary study on integrated wireless smart terminals for leaf area index measurement. Comput. Electron. Agric. 129, 56–65. doi: 10.1016/j.compag.2016.09.011

[B41] Ran Z. Xu Y. (2009). Entropy and weak solutions in the thermal model for the compressible Euler equations. Int. J. Modern Phys. C 20 (10), 1493–1519. doi: 10.1142/S0129183109014369

[B42] Raupach M. R. Shaw R. H. (1982). Averaging procedures for flow within vegetation canopies. Boundary-layer meteorology 22 (1), 79–90. doi: 10.1007/BF00128057

[B43] Reichard D. L. Fox R. D. Brazee R. D. Hall F. R. (1979). Air velocities delivered by orchard air sprayers. Trans. ASAE 22 (1), 69–0074. doi: 10.13031/2013.34968

[B44] Tang Q. Zhang R. R. Chen L. P. Li L. L. Xu G. (2021). Research progress of key technologies and verification methods of numerical modeling for plant protection unmanned aerial vehicle application. Smart Agric. 3 (3), 1. doi: 10.12133/j.smartag.2021.3.3.202107-SA004

[B45] Tudi M. Daniel Ruan H. Wang L. Lyu J. Sadler R. Connell D.. (2021). Agriculture development, pesticide application and its impact on the environment. Int. J. Environ. Res. Public Health 18 (3), 1112. doi: 10.3390/ijerph18031112 33513796 PMC7908628

[B46] Vollsinger S. Mitchell S. J. Byrne K. E. Novak M. D. Rudnicki M. (2005). Wind tunnel measurements of crown streamlining and drag relationships for several hardwood species. Can. J. For. Res. 35 (5), 1238–1249. doi: 10.1139/x05-051

[B47] Weickert M. Teike G. Schmidt O. Sommerfeld M. (2010). Investigation of the LES WALE turbulence model within the lattice Boltzmann framework. Comput. Mathematics Appl. 59 (7), 2200–2214. doi: 10.1016/j.camwa.2009.08.060

[B48] Wilson N. R. Shaw R. H. (1977). A higher order closure model for canopy flow. J. Appl. Meteorology (1962-1982) 16 (11), 1197–1205. doi: 10.1175/1520-0450(1977

[B49] Xun L. Garcia-Ruiz F. Fabregas F. X. Gil E. (2022). Pesticide dose based on canopy characteristics in apple trees: Reducing environmental risk by reducing the amount of pesticide while maintaining pest and disease control efficacy. Sci. Total Environ. 826, 154204. doi: 10.1016/j.scitotenv.2022.154204 35235850

[B50] Zhang H. Qi L. Wan J. Musiu E. M. Zhou J. Lu Z.. (2022). Numerical simulation of downwash airflow distribution inside tree canopies of an apple orchard from a multirotor unmanned aerial vehicle (UAV) sprayer. Comput. Electron. Agric. 195, 106817. doi: 10.1016/j.compag.2022.106817

[B51] Zhang H. Qi L. Wu Y. Musiu E. M. Cheng Z. Wang P. (2020). Numerical simulation of airflow field from a six–rotor plant protection drone using lattice Boltzmann method. Biosyst. Eng. 197, 336–351. doi: 10.1016/j.biosystemseng.2020.07.018

[B52] Zhang B. Tang Q. Chen L. P. Zhang R. R. Xu M. (2018). Numerical simulation of spray drift and deposition from a crop spraying aircraft using a CFD approach. Biosyst. Eng. 166, 184–199. doi: 10.1016/j.biosystemseng.2017.11.017

[B53] Zhu Y. Guo Q. Tang Y. Zhu X. He Y. Huang H.. (2022). CFD simulation and measurement of the downwash airflow of a quadrotor plant protection UAV during operation. Comput. Electron. Agric. 201, 107286. doi: 10.1016/j.compag.2022.107286

[B54] Zhu J. J. Matsuzaki T. Gonda Y. (2003). Optical stratification porosity as a measure of vertical canopy structure in a Japanese coastal forest. For. Ecol. Manage. 173 (1-3), 89–104. doi: 10.1016/S0378-1127(01)00813-1

